# Action of Varespladib (LY-315920), a Phospholipase A_2_ Inhibitor, on the Enzymatic, Coagulant and Haemorrhagic Activities of *Lachesis muta rhombeata* (South-American Bushmaster) Venom

**DOI:** 10.3389/fphar.2021.812295

**Published:** 2022-01-12

**Authors:** Pamella G. Gutierres, Diego R. Pereira, Nataly L. Vieira, Lilian F. Arantes, Nelson J. Silva, Kristian A. Torres-Bonilla, Stephen Hyslop, Karen Morais-Zani, Rosa M. B. Nogueira, Edward G. Rowan, Rafael S. Floriano

**Affiliations:** ^1^ Laboratory of Toxinology and Cardiovascular Research, University of Western São Paulo, Presidente Prudente, Brazil; ^2^ Graduate Program in Zootechnics, Rural Federal University of Pernambuco, Recife, Brazil; ^3^ Graduate Program in Environmental Sciences and Health, School of Medical, Pharmaceutical and Biomedical Sciences, Pontifical Catholic University of Goiás, Goiânia, Brazil; ^4^ Department of Pharmacology, Faculty of Medical Sciences, State University of Campinas, Campinas, Brazil; ^5^ Laboratory of Herpetology, Butantan Institute, São Paulo, Brazil; ^6^ Strathclyde Institute of Pharmacy and Biomedical Sciences, University of Strathclyde, Glasgow, United Kingdom

**Keywords:** Viperidae snake, phospholipase A2 (PLA2), coagulating activity, haemorrhage, varespladib, antivenom, neutralization

## Abstract

Varespladib (VPL) was primarily developed to treat inflammatory disturbances associated with high levels of serum phospholipase A_2_ (PLA_2_). VPL has also demonstrated to be a potential antivenom support agent to prevent PLA_2_-dependent effects produced by snake venoms. In this study, we examined the action of VPL on the coagulant, haemorrhagic and enzymatic activities of *Lachesis muta rhombeata* (South-American bushmaster) venom. Conventional colorimetric enzymatic assays were performed for PLA_2_, caseinolytic and esterasic activities; *in vitro* coagulant activities for prothrombin time (PT) and activated partial thromboplastin time (aPTT) were performed in rat citrated plasma through a quick timer coagulometer, whereas the dimensions of haemorrhagic haloes obtained after i.d. injections of venom in Wistar rats were determined using ImageJ software. Venom (1 mg/ml) exhibited accentuated enzymatic activities for proteases and PLA_2_ *in vitro*, with VPL abolishing the PLA_2_ activity from 0.01 mM; VPL did not affect caseinolytic and esterasic activities at any tested concentrations (0.001–1 mM). In rat citrated plasma *in vitro*, VPL (1 mM) alone efficiently prevented the venom (1 mg/ml)-induced procoagulant disorder associated to extrinsic (PT) pathway, whereas its association with a commercial antivenom successfully prevented changes in both intrinsic (aPTT) and extrinsic (PT) pathways; commercial antivenom by itself failed to avoid the procoagulant disorders by this venom. Venom (0.5 mg/kg)-induced hemorrhagic activity was slightly reduced by VPL (1 mM) alone or combined with antivenom (antivenom:venom ratio 1:3 ‘v/w’) in rats, with antivenom alone producing no protective action on this parameter. In conclusion, VPL does not inhibit other major enzymatic groups of *L. m. rhombeata* venom, with its high PLA_2_ antagonize activity efficaciously preventing the venom-induced coagulation disturbances.

## Introduction

Snakes of *Lachesis* genus are represented by three species found in Central America (*L. stenophrys*, *L. melanocephala*, and *L. acrochorda*) and one in South America (*L. muta*), with the latter being recognized as two subspecies distributed in the Amazon river basin (=*L. muta muta*) and Atlantic rainforest (=*L. muta rhombeata*) in Brazil (Costa and Bérnils, 2018; Nogueira et al., 2019; Diniz-Sousa et al., 2020). Together, these snakes are responsible by the second most frequent cases of snakebites in Americas, being exceeded only by *Bothrops* snakes (Magalhães et al., 2019; Diniz-Sousa et al., 2020).

Envenomation by *Lachesis* spp. is characterized by intense local pain, oedema and necrosis (Damico et al., 2006; Ferreira et al., 2009; Damico et al., 2012), systemic myotoxicity (Fuly et al., 2000; Fuly et al., 2003; Damico et al., 2006), renal failure (Damico et al., 2007), haemorrhage and coagulopathy (Sánchez et al., 1987; Sánchez et al., 1991; Sánchez et al., 1995; Fuly et al., 1997; Rucavado et al., 1999; Estêvão-Costa et al., 2000; Torres-Huaco et al., 2013), including severe cardiovascular disorders (Diniz and Oliveira, 1992; Giovanni-De-Simone et al., 1997; Dias et al., 2016a; Dias et al., 2016b). Such effects have been associated predominantly with the presence of phospholipases A_2_ (PLA_2_) (Cordeiro et al., 2015; Diniz-Sousa et al., 2018), metalloproteases (Cordeiro et al., 2018) and serine proteases (Wiezel et al., 2019), including biologically active peptides (Graham et al., 2005; Soares et al., 2005; Sanz et al., 2008; Pla et al., 2013; Pinheiro-Júnior et al., 2018), in these venoms.

Polyvalent antivenom (=anti-*Bothrops*/*Lachesis* serum) therapies comprise the main therapeutic options to treat the systemic envenomation by *Lachesis* snakes (Madrigal et al., 2017; Solano et al., 2018). Recently, several studies have demonstrated the value of varespladib, a PLA_2_ inhibitor drug (Lewin et al., 2016; Salvador et al., 2019), concerning its suppressive action on the biological effects of Elapidae and Viperidae venoms (Bittenbinder et al., 2018; Lewin et al., 2018; Wang et al., 2018; Zinenko et al., 2020; Gutiérrez et al., 2020a), including their toxins (Bryan-Quirós et al., 2019; Salvador et al., 2021). However, there are not reports about the action of varespladib, as a stand-alone therapy and/or combined with antivenoms, on the toxic effects caused by *Lachesis* venoms. In the present study, we have investigated the efficiency of this drug on some aspects of the envenomation by *Lachesis muta rhombeata* venom using *in vitro* and *in vivo* approaches for enzymatic, coagulant and haemorrhagic activities of this venom. We have also assessed an eventual synergic mechanism of action by varespladib when combined with a commercial antivenom used to treat envenomations by *Lachesis* in Brazil.

## Materials and Methods

### Reagents

Varespladib (LY-315920) was obtained from Sigma-Aldrich Chemical Co. (St. Louis, MO, United States) and anti-*Bothrops*/*Lachesis* serum was from Butantan Institute (São Paulo, SP, Brazil); varespladib was dissolved in DMSO prior to use, whereas the antivenom was provided ready for injection and maintained under refrigeration. Azocasein (A2765), Nα-Benzoyl-dl-arginine 4-nitroanilide hydrochloride (B4875) and 4-nitro-3-octanoyloxy-benzoic acid (N1646) substrates were also from Sigma-Aldrich Chemical Co. (St. Louis, MO, United States). *Lachesis m. rhombeata* venom was provided by Center for Biological Studies and Research of the Pontifical Catholic University of Goiás (PUC Goiás, Goiânia, GO, Brazil) through Dr Nelson J. Silva Jr. A lyophilized pool of venom obtained from one female adult snake was stored at −20°C and dissolved in ultrapure water prior to use.

### Animals

Wistar rats (300–350 g; 2–3 months old) obtained from Central Bioterium of the University of Western São Paulo (UNOESTE, Presidente Prudente, SP, Brazil) were housed in plastic cages (3 animals/cage) with a wood-shaving substrate, at 23 ± 1°C on a 12-h light/dark cycle with lights on at 6 a.m. The animals had free access to food and water. The experimental procedures were approved by an institutional Committee for Ethics in Animal Use (CEUA/UNOESTE, Protocol No. 6713/2021) and were done according to the general ethical guidelines for animal use established by the Brazilian Society of Laboratory Animal Science (SBCAL) and Brazilian Federal Law No. 11.794 of October 8, 2008, in conjunction with the guidelines for animal experiments established by the Brazilian National Council for Animal Experimentation (CONCEA).

### Phospholipase A_2_ (PLA_2_) Activity

PLA_2_ activity was assayed essentially as described elsewhere (Carregari et al., 2013). The standard assay mixture contained 200 μl of buffer (10 mM Tris-HCl, 10 mM CaCl_2_ and 100 mM NaCl, pH 8.0), 20 μl of substrate (3 mM 4-nitro-3-octanoyloxy-benzoic acid) and 20 μl of sample [venom alone (1 mg/ml) or venom (1 mg/ml) pre-incubated (for 30 min at 37°C) with varespladib (0.001–1 mM)] in a final volume of 240 μl. After adding sample, the mixture was incubated for 30 min at 37°C, with one unit of enzymatic activity being defined as an increase in absorbance of 0.001/min at 425 nm. All assays were done in triplicate with readings at 60-s intervals using a SpectraMax 340 multiwell plate reader (Molecular Devices, San Jose, CA, United States).

### Caseinolytic Activity

Caseinolytic activity was determined through colorimetric assay in a SpectraMax 340 multiwell plate reader (Molecular Devices, San Jose, CA, United States) using Azocasein as substrate, essentially as described elsewhere (Torres-Bonilla et al., 2020). The standard assay mixture contained 90 μl of substrate (212 mM Azocasein), 10 μl of reaction buffer (0.05 M Tris-HCl, 1 mM CaCl_2_, pH 8.0) and 10 μl of sample [venom alone (1 mg/ml) or venom (1 mg/ml) pre-incubated (for 30 min at 37°C) with varespladib (0.001–1 mM)] in a final volume of 110 μl. The mixture was incubated for 90 min at 37°C and then the reaction was terminated adding 200 μl of TCA 5% for 5 min at room temperature; the mixture was centrifuged (5 min at 8.000 g) and 150 μl of supernatant was transferred to the multiwell plate containing the same volume of NaOH (0.5 M). Finally, the absorbance was read at 440 nm via endpoint mode, with one activity unit being defined as an increase of absorbance of 0.001/min.

### Esterase Activity

Esterase activity was assayed essentially as described by (Erlanger et al., 1961) and adapted by (Torres-Bonilla et al., 2020). The standard assay mixture contained 200 μl of substrate (100 mM Nα-Benzoyl-dl-arginine 4-nitroanilide hydrochloride), 50 μl of reaction buffer (10 mM Tris-HCl, 10 mM CaCl_2_, 100 mM NaCl, pH 8.0), 15 μl of ultrapure water and 5 μl of sample [venom alone (1 mg/ml) or venom (1 mg/ml) pre-incubated (for 30 min at 37°C) with varespladib (0.001–1 mM)] in a final volume of 270 μl. The mixture was incubated for 30 min at 37°C in a multiwell plate and then read under an absorbance at *λ* = 410 nm via endpoint mode, with one activity unit being defined as an increase of absorbance of 0.001/min.

### Coagulant Activity

Coagulant activity was performed using Labtest^®^ commercial kits (Labtest Diagnóstica S.A., Lagoa Santa, MG, Brasil) performed in a quick timer Coagmaster 4.0 (Wama Diagnóstica Produtos para Laboratórios, São Carlos, SP, Brazil). Wistar rats were anesthetized by a non-lethal dose (1.8 mg/kg, i.p.) of thiopental (Cristália^®^, São Paulo, SP, Brazil) and, subsequently, subjected to intracardiac puncture in order to obtain blood samples using BD Vacutainer^®^ Citrate Tubes with 3.2% buffered sodium citrate solution at an anticoagulant:blood ratio of 1:10 (v/v); after this procedure, the animals were euthanased in saturated atmosphere with CO_2_. Blood samples were centrifuged (2,500 g, 4°C, 15 min) in order to obtain citrated platelet-poor plasma used in the prothrombin time (PT) and activated partial thromboplastin clotting time (aPTT) assays at 37°C; for each assay, 190 μl of platelet-poor plasma was incubated at 37°C with 10 μl of sample [1 – saline solution, 2 – varespladib (1 mM) or 3 – antivenom (antivenom:venom ratio 1:3 ‘v/w’), 4 – *L. m. rhombeata* venom (1 mg/ml), 5 – *L. m. rhombeata* venom + VPL, 6 – *L. m. rhombeata* venom + antivenom, 7 – and *L. m. rhombeata* venom + VPL + antivenom]. Antivenom:venom ratio was based on the manufacturer’s stated neutralizing capacity for the antivenom, where 1 ml of antivenom neutralizes 3 mg of *L. muta* venom (Instituto Butantan, São Paulo, SP, Brazil). The minimum effective dose of varespladib (1 mM) was confirmed in pilot experiments. Protocols: 1 − *L. m. rhombeata* venom was pre-incubated with VPL and/or antivenom for 30 min at 37°C before PT- and aPTT-required clot formation recording; 2 − *L. m. rhombeata* venom was directly exposed to VPL and/or antivenom and followed by immediate PT- and aPTT-required clot formation recording.

### Haemorrhagic Activity

Hemorrhagic activity was performed according to (Theakston and Reid, 1983). Male Wistar rats were anesthetized with sodium thiopental (1.8 mg/kg, i.p.) and their dorsal region was trichotomized in order to set the injection sites. Initially, some doses of *L. m. rhombeata* venom (0.01, 0.05, 0.1, 0.5 and 1 mg/kg) were tested in order to find the minimum haemorrhagic dose (0.5 mg/kg), which was used to investigate the neutralizing action of antivenom (antivenom:venom ratio of 1:3 ‘v/w’) and varespladib (1 mM). Protocol: after injection of venom (i.d.), the animals were subsequently treated with antivenom and/or varespladib via an intraperitoneal injection, followed by monitoring period of 24 h. Control sites were injected with 0.9% NaCl, DMSO (varespladib solvent) or antivenom. After 24 h, the rats were euthanized in saturated atmosphere with CO_2_, the dorsal skin was removed and the subcutaneous hemorrhagic halos in the inner surface were measured through ImageJ software (National Institute of Health, Bethesda, Maryland, United States).

### Statistical Analysis

All results (enzymatic, coagulant and haemorrhagic) were expressed as the mean ± SDM and statistical comparisons were done using Student’s *t* test or ANOVA followed by the Tukey–Kramer test, with *p* < 0.05 indicating significance. Data were analyzed using SAS University Edition software (SAS Institute Inc., Cary, NC, United States).

## Results

### Inhibitory Action of Varespladib on the Main Enzymatic Groups of *L. m. rhombeata* Venom

**FIGURE 1:**
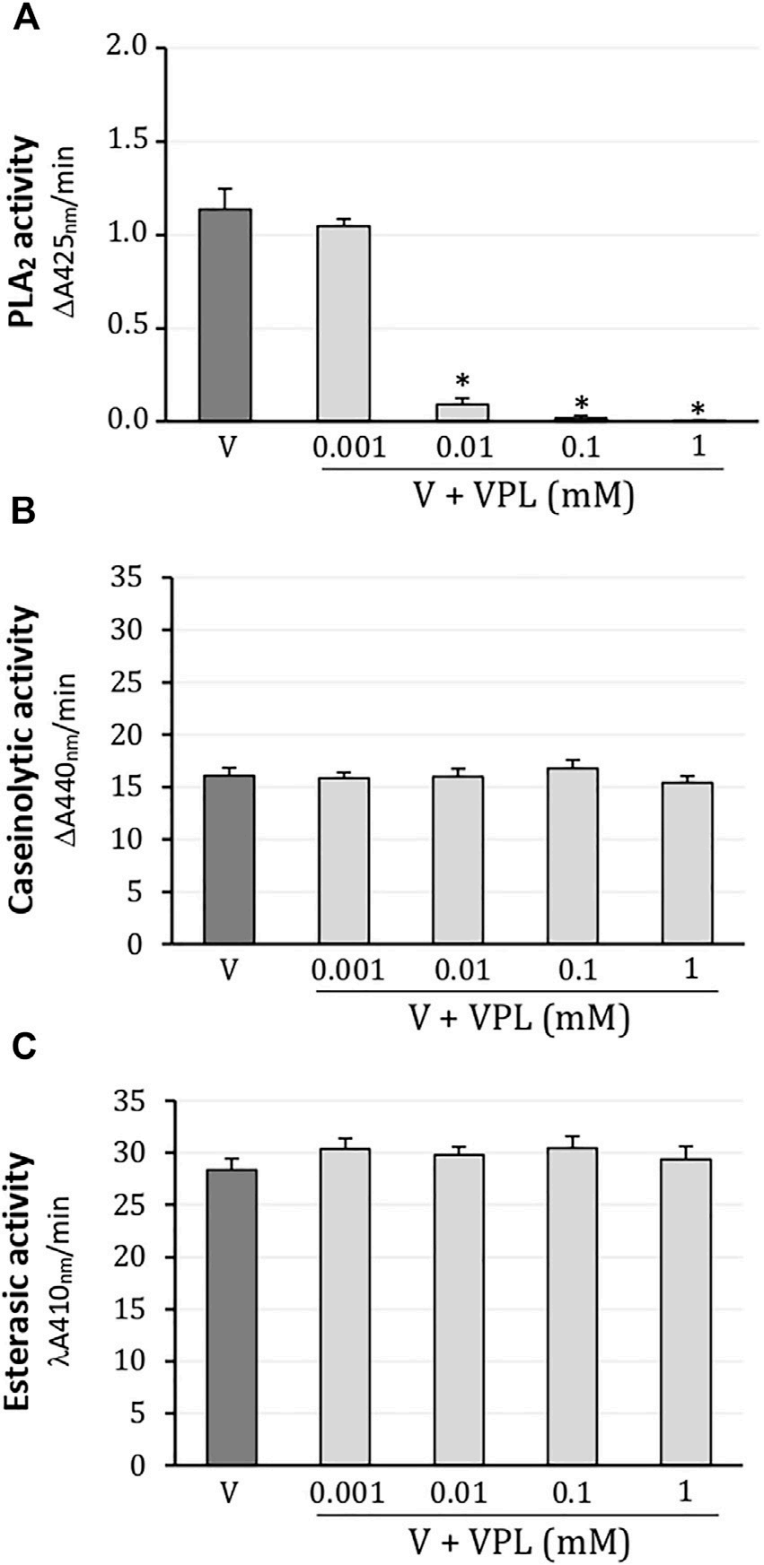
Action of varespladib on the major enzymatic activities of *L. m. rhombeata* venom. **(A)** Varespladib abolished the PLA_2_ activity at low concentrations. Unaffected **(B)** caseinolytic and **(C)** esterasic activities by varespladib at any tested concentration. Bars represent the mean ± SDM (*n* = 5). *p < 0.05 compared to venom alone.

In colorimetric assays, *L. m. rhombeata* (1 mg/ml) exhibited moderate enzymatic activity for PLA_2_ which was promptly abolished from 0.01 mM of varespladib; the lowest concentration of varespladib (0.001 mM) did not produce inhibitory effect on the PLA_2_ activity of this venom (Figure 1A). Caseinolytic (Figure 1B) and esterasic (Figure 1C) activities were not affected by any of these concentrations of varespladib tested on artificial substrates (0.001–1 mM).

### Inhibitory Action of Varespladib on the Coagulant Effect of *L. m. rhombeata* Venom in Rat Citrated Plasma

**FIGURE 2:**
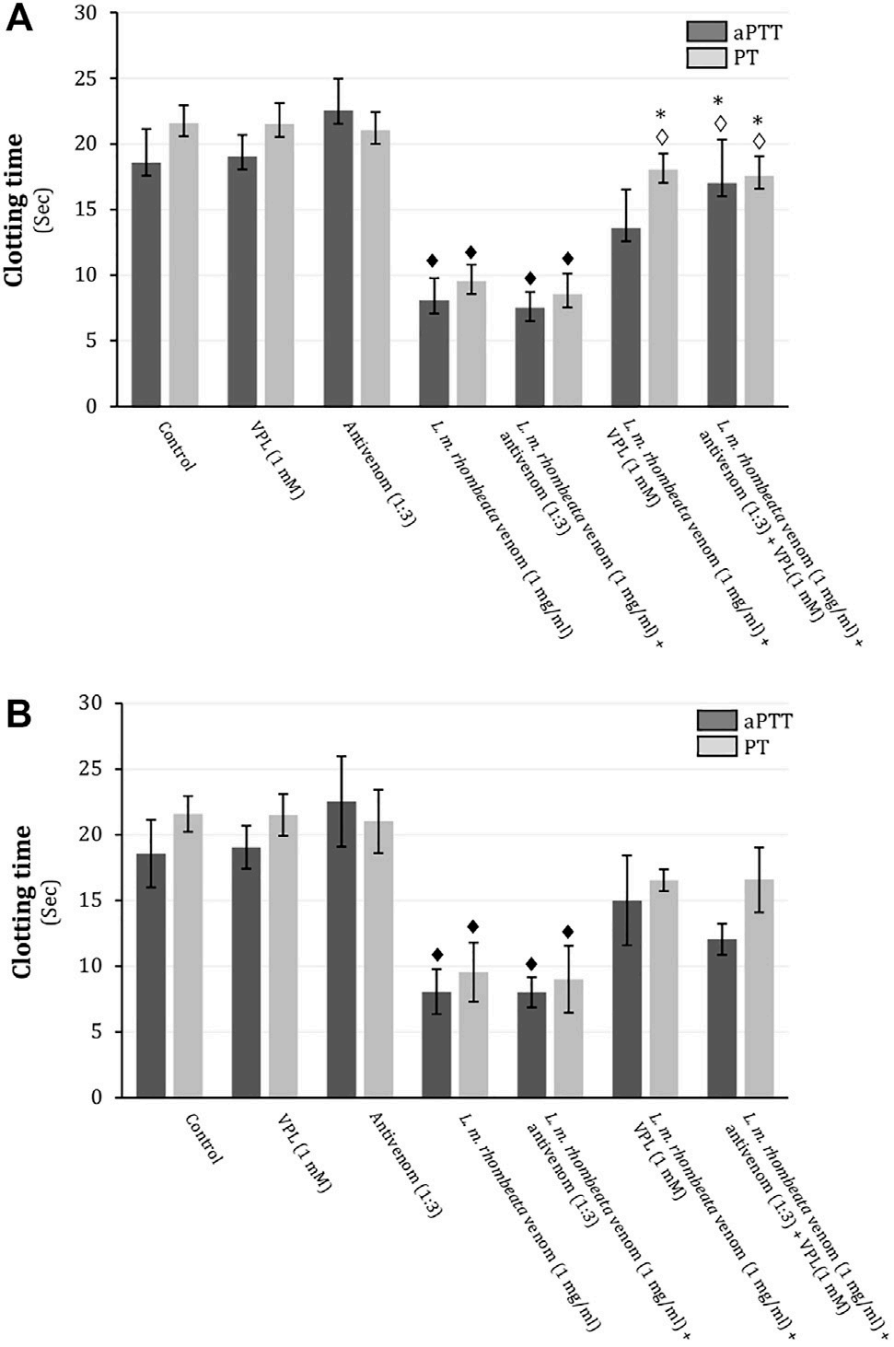
Action of varespladib on the procoagulant activity of *L. m. rhombeata* venom in rat citrated plasma *in vitro*. **(A)** Antivenom did not prevent the venom-induced procoagulant action applying the pre-incubation protocol, whereas varespladib alone avoided the procoagulant action for PT and its association with antivenom successfully prevented both intrinsic and extrinsic disorders. **(B)** Antivenom did not prevent the venom-induced procoagulant action applying the direct exposure protocol, whereas varespladib alone or combined with antivenom produced minor protective action on both coagulant pathways even combined with antivenom. Bars represent the mean ± SDM (*n* = 6). ^♦^ *p* < 0.05 compared to control, ^◊^ *p* < 0.05 compared to venom alone and *p < 0.05 compared to antivenom alone.

In rat citrated plasma, *L. m. rhombeata* venom (1 mg/ml) exhibited procoagulant action on the aPPT (intrinsic pathway) and PT (extrinsic pathway), decreasing in approximately 56.5 and 55.7% these times, respectively (*p* < 0.05 compared to basal values for both, *n* = 6) (Figures 2A,B). *L. m. rhombeata* venom (1 mg/ml)-induced procoagulant action (aPTT and PT) was not prevented by pre-incubating venom with antivenom (antivenom:venom ratio of 1:3 ‘v/w’) alone for 30 min at 37°C before clotting assay; however, varespladib (1 mM) alone significantly prevented the venom-induced procoagulant action for PT, with approximately 16.4% of decreasing being verified (*p* < 0.05 compared to venom alone, *n* = 6), and it partially avoided the venom-induced procoagulant action for aPTT, being observed approximately 26.9% of decreasing; the combination of both agents successfully contributed to avoid both intrinsic and extrinsic disorders in rat citrated plasma, resulting in 8.4 and 18.6% of decreasing for PT and aPTT, respectively (*p* < 0.05 compared to venom alone, *n* = 6) (Figure 2A). In assays performed immediately after the exposure *L. m. rhombeata* venom (1 mg/ml) to antivenom (antivenom:venom ratio of 1:3 ‘v/w’) and/or varespladib (1 mM), both of agents slightly delayed the *L. m. rhombeata* venom (1 mg/ml)-induced procoagulant action for PT and aPTT; under this experimental condition, varespladib alone or combined with antivenom were more effective in avoiding only the venom-induced procoagulant action for PT, with approximately 23.3% of decreasing being verified using both agents (Figure 2B).

### Inhibitory Action of Varespladib on the *L. m. rhombeata* Venom-Induced Subcutaneous Haemorrhage in Rat

**FIGURE 3:**
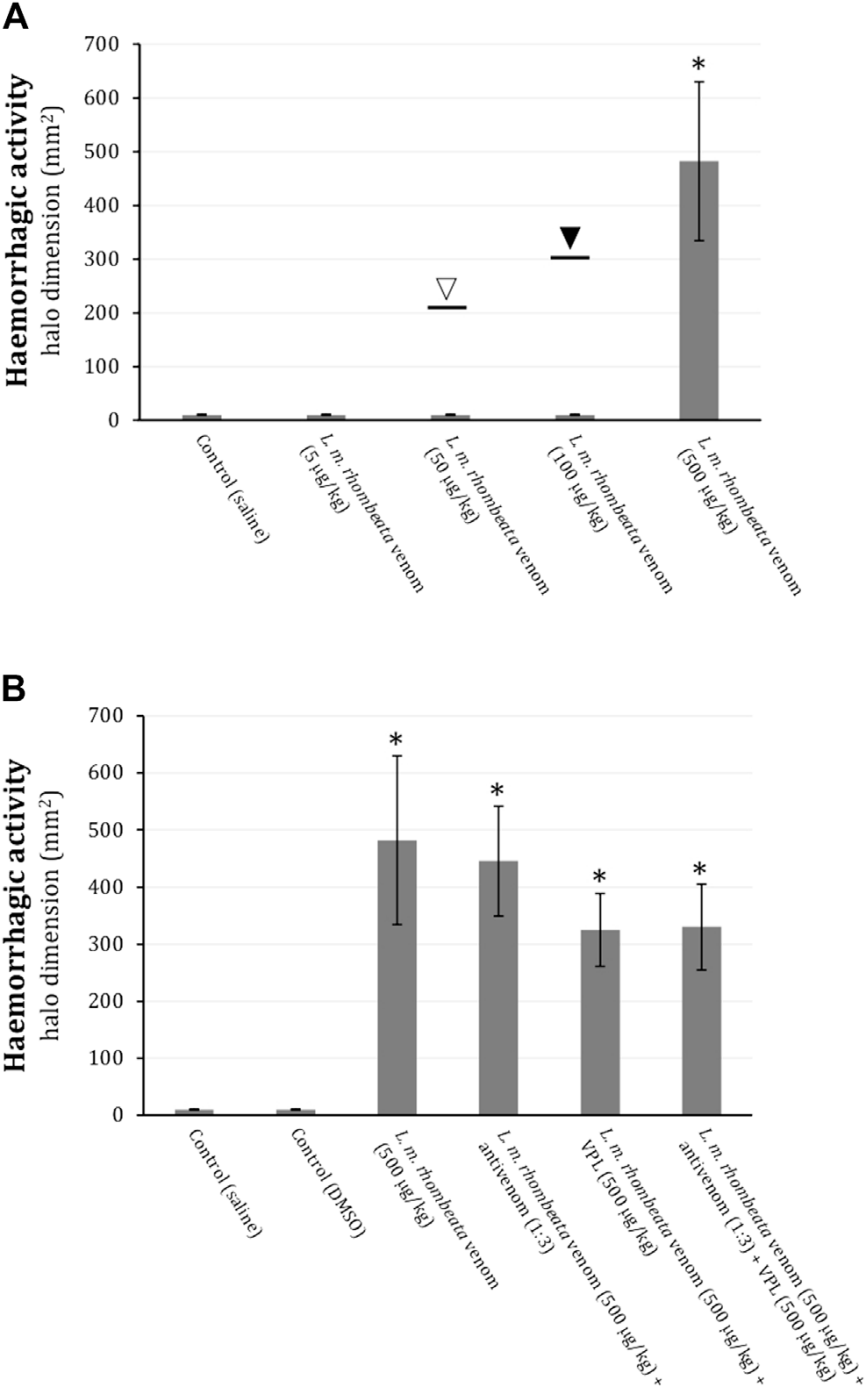
Action of varespladib on the haemorrhagic activity of *L. m. rhombeata* venom in rats. **(A)** *L. m rhombeata* venom produced intense subcutaneous haemorrhage at a dose of 500 μg of venom/kg, with the lowest doses (5, 50, and 100 μg/kg) being ineffective to produce haemorrhage; the opened and filled arrows indicate the haemorrhagic dimension caused in single animals envenomed with 50 and 100 μg of venom/kg, respectively. **(B)** Antivenom did not prevent the venom-induced subcutaneous haemorrhage, with varespladib alone or combined with antivenom producing discreet reduction of this alteration. Bars represent the mean ± SDM (*n* = 6). *p < 0.05 compared to control (saline).

*L. m. rhombeata* venom at low doses (5, 50 and 100 μg/kg) did not produce subcutaneous haemorrhagic action in rats, however, with exceptionally one animal exhibiting ∼213 mm^2^ of haemorrhagic halo for 50 μg of venom/kg (opened arrow) and another one ∼301 mm^2^ for 100 μg of venom/kg (filled arrow); the higher dose of this venom (500 μg/kg) induced pronounced haemorrhagic halo formation (*p* < 0.05 compared to control saline, n = 6) (Figure 3A). Antivenom (antivenom:venom ratio of 1:3 ‘v/w’) administered (i.p) immediately after intradermic injections of *L. m. rhombeata* venom (500 μg/kg) was not able to prevent the venom-induced subcutaneous haemorrhage; varespladib (1 mM) alone or associated with antivenom (antivenom:venom ratio of 1:3 ‘v/w’) exhibited a mild protective effect on the venom-induced subcutaneous haemorrhage (Figure 3B).

## Discussion

Envenomations by Viperidae snakes comprise a serious public health problem in Latin America (Chippaux, 2017; Ochoa-Avilés et al., 2020; Gutiérrez et al., 2020b). In Brazil, these snakes are represented by three main genera, i.e., *Bothrops*, *Crotalus* and *Lachesis* (Viperidae–Crotalinae), being responsible for more than 20,000 cases of snakebites per year in this country, as reported by Notifiable Diseases Information System of the Brazilian Ministry of Health (SINAN, Brasília, DF, Brazil). *Lachesis* snakes found in South America (*L. muta muta* and *L. m. rhombeata*) occasionally cause severe human envenomations (Magalhães et al., 2019; Diniz-Sousa et al., 2020), which are characterized by pronounced local and systemic disorders, e.g., necrosis (Damico et al., 2006; Ferreira et al., 2009; Damico et al., 2012), haemorrhage, coagulopathy (Sánchez et al., 1987; Sánchez et al., 1991; Sánchez et al., 1995; Fuly et al., 1997; Rucavado et al., 1999; Estêvão-Costa et al., 2000; Torres-Huaco et al., 2013) and hypotension (Dias et al., 2016a; Dias et al., 2016b), strongly associated with a variety of enzymatically active proteins such as snake venom metalloproteases, serine proteases, PLA_2_, C-type lectins and l-amino acid oxidases (Weinberg et al., 2004; Junqueira-de-Azevedo et al., 2006; Bregge-Silva et al., 2012; Madrigal et al., 2012; Cordeiro et al., 2018; Diniz-Sousa et al., 2018; Wiezel et al., 2019) present in these venoms.

In recent years, the PLA_2_ antagonistic activity of varespladib (Lewin et al., 2016; Salvador et al., 2019; Salvador et al., 2021), a synthetic drug developed to treat disturbances of inflammatory cascades associated with high levels of secreted PLA_2_ (Varespladib, 2011), has been experimentally explored as an useful therapeutic alternative to complement antivenom therapies applied in envenomations by Elapidae and Viperidae snakes, with potential even to replace them in special situations in which these antivenoms are not available. Varespladib has high efficacy to suppress the systemic effects caused by several venoms from Elapidae (Lewin et al., 2016; Bittenbinder et al., 2018; Lewin et al., 2018; Wang et al., 2018; Oliveira et al., 2020; Gutiérrez et al., 2020a; Dashevsky et al., 2021; Kazandjian et al., 2021; Silva-Carvalho et al., 2021) and Viperidae (Lewin et al., 2016; Wang et al., 2018; Youngman et al., 2020; Zinenko et al., 2020; Gutiérrez et al., 2020a; Liu et al., 2021) snakes. However, there is no report associating the efficacy of varespladib with toxicological aspects of *Lachesis* venoms.

Based on this premise, we have unprecedentedly investigated the action of varespladib on the enzymatic, coagulant and haemorrhagic activities of *Lachesis muta rhombeata* venom to determine its efficiency as a single pharmacological tool or combined with a commercial antivenom used in Brazil. In summary, we have demonstrated that varespladib used as a single pharmacological tool abolishes the PLA_2_ activity of *L. m. rhombeata* venom at low concentrations, without affecting the catalytic activity for proteases (metalloprotease and serino protease) of the venom, indicating a very specific inhibitory activity; its high PLA_2_ antagonistic activity was reflected on the venom-induced procoagulant action, mostly interfering on the extrinsic pathway disorders produced by venom in rat citrated serum (pre-incubation protocol), whereas the drug did not prevent the haemorrhagic activity induced by *L. m. rhombeata* venom in rats. The association of varespladib with a commercial antivenom used in Brazil to treat envenomations by *Lachesis* spp. did not produce important synergic actions on the procoagulant (direct incubation protocol) and haemorrhagic effects induced by *L. m. rhombeata* venom; such interaction resulted in a major prevention of the venom-induced intrinsic and extrinsic coagulant disorders seen under pre-incubation protocol.

Although the procoagulant action of Viperidae venoms has been mostly associated with the presence of serine proteases in these venoms (Gutiérrez et al., 2021), varespladib can partially prevent the procoagulant action of *L. m. rhombeata* venom, indicating an eventual role of PLA_2_ in these processes. Accordingly, varespladib also contributes to prevent coagulating disorders induced by other groups of snake venoms, e.g., *Bothrops* (Viperidae-Crotalinae), *Daboia*, *Echis*, *Oxyuranus*, *Naja*, *Pseudechis* and *Bitis* spp., which exhibit high PLA_2_ activity (Bittenbinder et al., 2018; Xie et al., 2020; Youngman et al., 2020; Zdenek et al., 2020). On the other hand, varespladib does not affect the *L. m. rhombeata* venom-induced subcutaneous haemorrhage in rats, reflecting its disability in antagonizing the metalloproteases of this venom, since local and systemic haemorrhagic actions produced by Viperidae venoms are mainly mediated by this family of toxins (Escalante et al., 2011; Seo et al., 2017). In addition, although an Asp49 PLA_2_ (LmrTX) with anticoagulant activity has been already isolated from *L. m. rhombeata* venom (Damico et al., 2012), it does not appear to contribute expressively with the subcutaneous haemorrhage induced in rats, as reported in this study.

*L. m. rhombeata* venom has been an important object of study for structural characterization of toxins such as metalloproteases (Cordeiro et al., 2018), serine proteases (Aguiar et al., 1996; Wiezel et al., 2019), C-type lectins (Wiezel et al., 2019), basic and acid PLA_2_ (Damico et al., 2012; Cordeiro et al., 2015; Diniz-Sousa et al., 2018), phospholipase B and hialuronidase (Wiezel et al., 2015), including bradykinin-potentiating peptides (BPPs) (Pinheiro-Júnior et al., 2018). As previously commented, these toxins are responsible by developing the main toxicological aspects of the envenomation by *Lachesis*. However, the factual involvement of PLA_2_ toxins on the effects produced by *Lachesis* venoms have been poorly investigated, with a few reports describing their inhibitory action of platelet aggregation (Cordeiro et al., 2015), anticoagulant and antithrombotic activities (Damico et al., 2012), and cytotoxicity on C2C12 myotubes (Diniz-Sousa et al., 2018).

In Brazil, envenomations by *L. m. muta* and *L. m. rhombeata* are treated with anti-*Bothrops*/*Lachesis* serums, with their rescue action being dependent on early i.v. administration (Pla et al., 2013; Madrigal et al., 2017; Solano et al., 2018). There are some negative factors that resulting in deaths by accidents involving *Lachesis* snakes in Brazil: 1) limited availability of antivenoms, 2) difficulties in accessing health services in certain regions of the country, and 3) lack of a specific anti-*Lachesis* serum. Consequently, other types of antivenoms, e.g., anti-*Bothrops* serum and anti-*Bothrops*/*Crotalus* serum, have been inaccurately recommended to treat envenomations by *L. muta* in the absence of anti-*Bothrops*/*Lachesis* serum (Magalhães et al., 2019; Muniz et al., 2021). These challenges involving the treatment of envenomations by *Lachesis* snakes strengthen the search for therapeutically useful adjuncts, with varespladib rising as a plausible tool.

In conclusion, varespladib abolishes efficiently the PLA_2_ activity of *L. m. rhombeata* venom at low concentrations and does not affect other majority enzymatic groups of this venom, e.g., metalloproteases and serine proteases. Varespladib alone might partially prevent the procoagulant effect of *L. m. rhombeata* venom, with its combination with antivenom avoiding alterations in both intrinsic and extrinsic pathways. Varespladib does not reduce the subcutaneous haemorrhage formation induced by *L. m. rhombeata* venom in rats due to the lack of inhibitory action on the proteases of the venom. The association of varespladib with a recommended polyvalent antivenom does not produce synergic action on the venom-induced haemorrhagic action but it helps to prevent the venom-induced coagulation disorders.

## Data Availability

The original contributions presented in the study are included in the Article/Supplementary Material, further inquiries can be directed to the corresponding author.
